# Suppression of cardiomyocyte functions by β-CTX isolated from the Thai king cobra (*Ophiophagus hannah*) venom via an alternative method

**DOI:** 10.1590/1678-9199-JVATITD-2020-0005

**Published:** 2020-07-17

**Authors:** Tuchakorn Lertwanakarn, Montamas Suntravat, Elda E. Sanchez, Worakan Boonhoh, R. John Solaro, Beata M. Wolska, Jody L. Martin, Pieter P. de Tombe, Kittipong Tachampa

**Affiliations:** 1 Department of Physiology, Faculty of Veterinary Science, Chulalongkorn University, Bangkok, Thailand.; 2 National Natural Toxins Research Center, Texas A&M University-Kingsville, Kingsville, TX, USA.; 3 Department of Chemistry, Texas A&M University-Kingsville, Kingsville, TX, USA.; 4 Department of Physiology and Biophysics, University of Illinois at Chicago, IL, USA.; 5 Department of Medicine, University of Illinois at Chicago, IL, USA.

**Keywords:** Beta-cardiotoxin, Cytotoxicity, Cardiomyocyte, King cobra, Purification

## Abstract

**Background:**

Beta-cardiotoxin (β-CTX), the three-finger toxin isolated from king cobra (*Ophiophagus hannah*) venom, possesses β-blocker activity as indicated by its negative chronotropy and its binding property to both β-1 and β-2 adrenergic receptors and has been proposed as a novel β-blocker candidate. Previously, β-CTX was isolated and purified by FPLC. Here, we present an alternative method to purify this toxin. In addition, we tested its cytotoxicity against different mammalian muscle cell types and determined the impact on cardiac function in isolated cardiac myocyte so as to provide insights into the pharmacological action of this protein.

**Methods:**

β-CTX was isolated from the crude venom of the Thai king cobra using reverse-phased and cation exchange HPLC. *In vitro* cellular viability MTT assays were performed on mouse myoblast (C2C12), rat smooth muscle (A7r5), and rat cardiac myoblast (H9c2) cells. Cell shortening and calcium transient dynamics were recorded on isolated rat cardiac myocytes over a range of β-CTX concentration.

**Results:**

Purified β-CTX was recovered from crude venom (0.53% w/w). MTT assays revealed 50% cytotoxicity on A7r5 cells at 9.41 ± 1.14 µM (n = 3), but no cytotoxicity on C2C12 and H9c2 cells up to 114.09 µM. β-CTX suppressed the extend of rat cardiac cell shortening in a dose-dependent manner; the half-maximal inhibition concentration was 95.97 ± 50.10 nM (n = 3). In addition, the rates of cell shortening and re-lengthening were decreased in β-CTX treated myocytes concomitant with a prolongation of the intracellular calcium transient decay, indicating depression of cardiac contractility secondary to altered cardiac calcium homeostasis.

**Conclusion:**

We present an alternative purification method for β-CTX from king cobra venom. We reveal cytotoxicity towards smooth muscle and depression of cardiac contractility by this protein. These data are useful to aid future development of pharmacological agents derived from β-CTX.

## Background

Beta-cardiotoxin (β-CTX) or cardiotoxin-27 (CTX27) is a member of three-finger toxins (3FTXs) isolated from the venom of the king cobra (*Ophiophagus hannah*). Purification of the protein was accomplished by size exclusion chromatography, followed by reverse phase C18 column separation using a fast-protein liquid chromatography (FPLC) system [1]. However, with regards to cost-efficiency, HPLC may provide an alternative method for purifying β-CTX as we explore here. The protein contains 63 amino acids with an estimated molecular weight of 7 kDa [1]. Like all cardiotoxins (CTXs), the protein is formed by a two beta-pleated structure, containing five small beta strands [2]. The toxin was previously found in the venom of the king cobra from various regions of Asia, including Malaysia, Indonesia, China, and Thailand [3-6]. The venoms of king cobra from different geographical regions have been found to be highly variable in their proteomes, including CTX abundances, and therefore the venom toxicity variations [6, 7]. 

The β-CTX yield from king cobra venom ranges between 0.2-2.0% w/w [4, 6]. However, the amount of β-CTX that can be recovered from the Thai snake has never been reported. Interestingly, β-CTX displays characteristics different from other CTXs. That is, a specific α-helical structure is revealed upon thermal unfolding of β-CTX consistent with its conformational plasticity. Despite overall structural similarity to conventional cardiotoxins, there are notable differences in both the loop region and overall surface charge distribution of the β-CTX protein that may underlie the relatively low cytosolic activity of this cardiotoxin compared to those more conventional cardiotoxins. Furthermore, the loss of charges distributed in the protein structure completely disregarded its cytolytic property [2]. β-CTX also shows negative chronotropy, dose-dependently, in both isolated heart and *in vivo* studies. The effect has been ascribed to antagonize β-agonist binding to both β-1 and -2 adrenergic receptors [1]. Hence, the compound has been proposed as a potential β-blocker candidate. Therefore, β-CTX is an interesting agent that can potentially be useful for development of novel pharmaceutical agents.

Prior to the introduction of β-CTX into drug development, the toxicology of the compound needs to be assessed. It has been reported that, at 100 mg/kg, β-CTX causes several neurological symptoms in mice, including respiratory distress, inability to move, unconsciousness and death 30 minutes following injection [1]. The same study also noted that the compound showed a non-lethal outcome up to 10 mg/kg. Moreover, β-CTX did not exhibit either anticoagulant or hemolytic effects [1]. However, *in vitro* cytotoxic studies have never been investigated. In addition, the direct effects of β-CTX at the cellular level, particularly on isolated cardiomyocyte function has not been reported. Therefore, the objectives of the current study were to develop an alternative simplified technique for purifying β-CTX from the Thai snake venom, to test the cytotoxicity on mammalian muscle cell lines, and to evaluate its effect on isolated cardiac myocyte function.

## Methods

### Snake venom, cell cultures, and animals

Lyophilized Thai king cobra venom (KCV) was purchased from Queen Saovabha Memorial Institute, Bangkok, Thailand. Mouse myoblast cell line C2C12 (ATCC^®^, CRL^TM^-1772), rat smooth muscle cell line A7r5 (Sigma Aldrich^®^, Cat No. 86050803), and rat cardiomyoblast H9c2 (ATCC^®^, CRL^TM^-1446) were cultured in Dulbecco’s Minimum Essential Medium (DMEM; ATCC^®^, 30-2002) with 10% fetal bovine serum, 50 U/mL of penicillin and 50 µg/mL of streptomycin. During cell growth and differentiation, the medium was changed every two days. The third to the fourth passages of cells were used for the *in vitro* cytotoxicity assay. Male adult Sprague Dawley rats (3-5 weeks old; 150-250 g) were used for the *ex vivo* isolated cardiomyocyte functional study.

### Isolation and identification of β-CTX

Lyophilized crude venom was reconstituted with 0.1% trifluoroacetic acid (TFA; Pierce^TM^, 28904) to make 50 mg/mL. Venom was then filtered using Acrodisc^®^ 0.45 µm GH Polypro (GHP) membrane (Life Sciences). Subsequently, 200 µL of filtered solution were applied to a Grace^®^ C18 column (Vydac 218TP^TM^; 5 µm, 300 Å, 4.6 mm. × i.d. 250 mm) which was pre-equilibrated with 0.1% v/v TFA in Milli Q water (solution A) and eluted with 80% v/v acetonitrile in 0.1% TFA (solution B). Flow rate of the binary HPLC system (Waters^®^ 1525) was set at 1 mL/min with linear gradients as follows: 5 minutes isocratic solution B (5%), followed by a linear gradients to 40% solution B over 95 minutes, a further gradient of 40-70% solution B over 20 minutes, ending with 10 minutes isocratic 70% solution B; following elution the column was re-equilibrated with solution A. Peaks were detected at 215 nm using an UV/Visible light detector (Waters^®^ 2489) and fractions were collected manually. Selected fractions, with the presence of β-CTX as confirmed by SDS-PAGE and N-terminal sequencing, were then pooled together, and lyophilized (FreeZone6; Labconco^®^).

Lyophilized fractions from RP-HPLC were reconstituted into 0.05 M sodium phosphate buffer, pH 7.4 and filtered. Subsequently, proteins were further sub-fractionated using a Waters^®^ cation exchange (cIEx) chromatography column (Protein Pak^TM^-SP 5 PW, 7.5 mm × i.d. 75 mm). The column was pre-equilibrated with 0.05 M sodium phosphate buffer, pH 7.4 (solution C) and eluted with 0.05 M sodium phosphate buffer, adding 0.5 M of sodium chloride, pH 7.4 (solution D); injection volume was 200 µL. The elution was performed over a 90 minutes gradient: 100% solution C isocratic for 5 minutes, followed by a linear gradient with solution D 0-70% over 70 minutes, isocratic hold at 70% solution D for 5 minutes, followed by a further 70-100% solution A gradient for 5 minutes; next the column was re-equilibrated to 100% solution C. A Waters^®^ 2489 Dual λ absorbance detector was used to detect proteins at 280 nm. Fractions were desalted using 100-500 Dalton MWCO cellulose ester dialysis membrane (Spectra/Por^®^) and concentrated by freeze-drying vacuum lyophilization at −40°C. The lyophilized β-CTX powder was kept at −80°C until further use.

### SDS-PAGE and automated N-terminal sequencing

Samples (5 µg protein) were loaded onto the gel as well as a standard protein marker (SeeBlue^TM^ plus2 pre-stained)**.** The gel employed was a precast 4-12% Bis-Tris gel (NuPAGE^®^; Novex) in an XCell SureLock™ Mini-Cell system (Invitrogen) and electrophoresed at 100 V for 95 minutes. Samples were run using 500 mM dithiothreitol (DTT, NuPAGE^®^) reducing agent. Next, gels were stained with SimplyBlue™ SafeStain (Life Technologies) and de-stained using Milli Q water. Following staining, eluted proteins were transferred onto a 0.45 µM polyvinylidene fluoride (PVDF) membrane (Immobilon^TM^-P; EMD Millipore^®^) using a Semi-Dry Transblot Cell system (Bio-Rad^®^) at 25 V for 1.5 hours. Bands were cut manually and loaded into an automated Edman degradation N-Terminal sequencer (PPSQ-33B; Shimadzu^®^). The first 14 amino acid residues were analyzed using commercial software (PPSQ-30 Analysis; Shimadzu^®^). Sequences of the acquired peptide were blast searched with the online website system (https://blast.ncbi.nlm.nih.gov/Blast.cgi?PAGE=Proteins&) so as to identify isolated proteins. Fractions with the confirmation of β-CTX were selected for further *in vitro* cytotoxicity and functional study of isolated cardiomyocytes.

### ***In vitro* cellular viability assay of β-CTX**

The MTT (methyl-thiazol-tetrazolium) assay was used to test cellular viability towards three different types of muscle cells. Briefly, cells in the culture flasks were harvested using 0.25% trypsin-EDTA solution (Sigma^®^, T4049). C2C12 (1.5 × 10^6^ cells/well), A7r5 (5 × 10^4^ cells/well) and H9c2 (1.5 × 10^4^ cells/well) were incubated at 37°C in 5% CO_2_ for 24 hours. The cells were treated with β-CTX-2 fraction (0.001-0.8 mg/mL; n = 3). Each experiment was carried out in triplicate. Changes in cell morphology of cells were identified using light microscopy with magnification 100x following 0 and 24 hours of incubation in the presence of toxin. Next, following 24 hours of culture, cells were exposed to MTT by the additions of 12 µL 0.5% MTT solution. After 4 hours, 100 µL dimethyl sulfoxide (DMSO) was added and Formazan color was detected at 570 nm using a plate reader (Beckman Coulter^TM^, AD340). Cells treated with sterile PBS were used as a negative control. 

### Isolation of adult rat cardiomyocytes

The ventricular myocytes isolation protocol was modified from Wolska and Solaro [8]. Briefly, the heart and attached aorta were cut from the rat thorax, weighed and cannulated onto a Langendorff apparatus. The heart was perfused retrograde with perfusion buffer (in mM: NaCl 133.5, KCl 4, NaH_2_PO_4_ 1.2, HEPES 10, MgSO_4_ 1.2, dextrose 33.33 and 0.1% BSA). The perfusion solution was then switched to enzyme solution (perfusion buffer containing: 0.025% collagenase II (Worthington^®^), 0.03% protease XIV (Sigma^®^) and 20 µM of CaCl_2_). After the digestion process, ventricles were cut and minced in perfusion buffer containing 50 µM CaCl_2_. The isolated cell containing solution was next filtered using a 100 µm nylon mesh and [Ca^2+^] was gradually reintroduced to up to 1 mM. Only isolation preparations with at least 70% surviving ventricular cells were used for further study. Quiescent and rectangular-shaped cells were next selected for the *ex vivo* isolated cardiac myocyte functional study.

### Measurement of isolated cardiac myocyte functional study

Following the cell isolation, cellular mechanical function and calcium homeostasis were assessed using the Photon Technology International (PTI^®^) system. Cells were incubated with Fura-2 AM (Thermo Fischer^®^, F1221), an intracellular calcium fluorophore, before placing them upon an inverted fluorescence microscope (Nikon^®^ Eclipse^TM^, TE-300). Cell contraction was measured and controlled by a video edge detector (Crescent electronics^®^, VED-105) and monitored with an oscilloscope (HAMEG^®^, HM205-3). The Xeon fluorescence lamp was set at 75-80 W. Calcium profiles were acquired by excitation of Fura-2 AM at 340 and 380 nm; fluorescent emission was recorded at 505 nm using fluorescence lamp source (DeltaRam X^TM^, PTI^®^). The signal was amplified by the photomultiplier tube (Model 814^TM^). 

Data were recorded using a data acquisition system (Digidata^®^ 1440a) employing compatible software (FeliX32 1.42B^TM^, PTI^®^). The captured data were analyzed using LabChart^TM^ 7 software. Isolated rat cardiac ventricular myocytes (3-5 replicates per rat) were measured first at baseline condition by perfusing with a control solution (perfusion buffer containing 1.8 mM CaCl_2_; n = 4). Next, cells were perfused with the same solution at which β-CTX was added over a range of concentrations (10 - 1000 nM) to determine the dose-response relationship. Recorded parameters were calculated as percentage change from baseline. Parameters analyzed in the study included: extend of cell shortening, shortening velocity (+dL/dt), relaxing index (τ), re-lengthening velocity (-dL/dt), Ca^2+^-transients amplitude (CaT) and Ca^2+^-decay time (τ_Ca_). Data were calculated from 8-10 consecutive cell contractions for each measurement condition. 

### Statistical analysis

A non-linear dose-response curve fit was applied to assess the cytotoxicity of β-CTX on both cell lines, expressed as the half-maximal cytotoxic concentration (CC_50_). Normal distributions of data were tested using the Kolmogorov-Smirnov test with Dallal-Wilkinson-Lillie for *p*-value. Likewise, non-linear curve fitting was used to assess the myocyte shortening, +dL/dt, τ, -dL/dt, CaT, and τ_Ca_ parameters. The half-maximal inhibitory concentration (IC_50_) was calculated from the myocyte shortening non-linear curve fit dose-response equation. All data are presented as mean ± S.E.M. All statistical analysis was performed using commercial software (GraphPad^®^ Prism). Repeated measure ANOVA was performed to evaluate the effect of β-CTX on each of the parameters. Dunnett’s method was used as a post-hoc analysis to compare between each concentration of the protein to the baseline. 

## Results

### Purification of β-CTX

**Figure 1.**
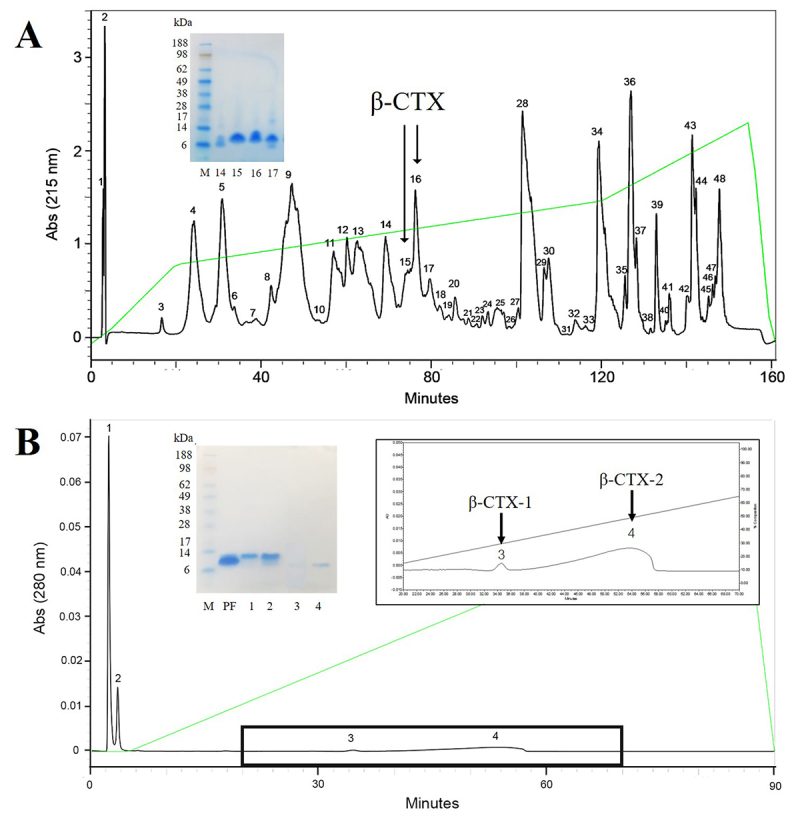
Chromatographic profiles of the β-CTX purification. **(A)** Reverse phase chromatographic profile represents fractions containing β-CTX (arrows), identified by SDS-PAGE (inset) and automated N-terminal sequencer. **(B)** Cation exchange HPLC profile of the pooled fractions (PF) 15-16th from the reverse phase HPLC. (Inset) β-CTX-1 and -2 were identified in both subfraction 3 and 4, respectively (arrows). All fractions were determined the protein constituents by running SDS-PAGE (inset) and N-terminal sequencer. M = protein marker (SeeBlue^TM^ Plus2, Thermo Fisher^®^).

Figure 1A shows chromatograms of 48 different peaks from KCV as fractionated by RP-HPLC. β-CTX was found in two different fractions (fractions 15 and 16 on the chromatogram; arrows) as confirmed by SDS-PAGE and N-terminal sequencing. The collected β-CTX-containing fractions were subsequently pooled and next subjected to a second purification step using cIEx chromatography. The cIEx chromatogram (Figure 1B) showed four different subfractions where both the 3^rd^ and 4^th^ fractions (Figure 1B; inset) contained a protein with an estimated molecular weight of 7.56 and 7.58 kDa, respectively. After confirming the amino acid sequence by automated N-terminal sequencer (Table 1), both β-CTX fractions were characterized and named as β-CTX-1 and -2, respectively (most likely isoforms of the toxin where the N-terminal G residue is replaced by R). As shown in Table 2, the final total yield of purified β-CTXs was approximately 0.53% of the crude KCV mass. However, in light of the larger amount of protein obtained from the isolation, only β-CTX-2 was further used for the cytotoxicity and functional studies. Note that we will refer to this fraction as β-CTX for the remainder of this report to highlight the notion that the observed cellular impacts likely apply to both isoforms. 

**Table 1.** N-terminal sequence results representing amino acid residues of β-CTX found from the isolation

RP-HPLC peak	Apparent mass (kDa)	Amino acid sequences (14 residues)	Identification	Accession No.
F15	7.78	GKPLNTPLPLIYYT	β-CTX	Q69CK0.1
F16	8.02	GKPLNTPLPLIYYT	β-CTX	Q69CK0.1
**cIEx Peak**	**Apparent mass (kDa)**	**Amino acid sequences (14 residues)**	**Identification**	**Accession No.**
F3	7.56	RKLLNTPLPLIYTT	β-CTX-1	Q69CK0.1
F4	7.58	GKLLNTPLPLIYT-	β-CTX-2	Q69CK0.1

**Table 2.** Purification profiles of β-CTX from Thai KCV

Purification step	Volume (mL)	Protein concentration (mg/mL)^a^	Total protein (mg)^b^	Recovery of protein (%)^c^
Crude KCV	4	100	200	100
C18-HPLC	4.71	2	9.42	4.71
SP cIEx				
**β-CTX-1**	0.14	1.2	0.17	0.08
**β-CTX-2**	0.36	2.5	0.9	0.45
Total			1.07	0.53

^a^ Protein concentration was obtained using a spectrophotometer at 280 nm.

^b^ Total protein was calculated by multiplying (total volume; mL) × (protein concentration; mg/mL).

^c^ Recovery of protein was defined as the total protein recovered from each purification step.

### ***In vitro* cellular viability of β-CTX on different muscle cell lines**

**Figure 2.**
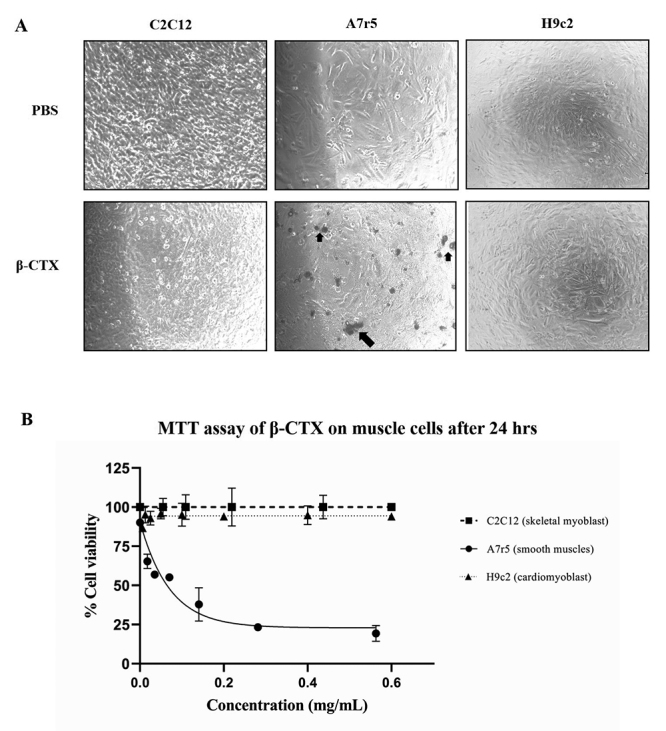
**(A)**Representative microscopic pictures of C2C12, A7r5 and H9c2 muscle cell lines treated with either PBS (top) or β-CTX (bottom). Concentration of β-CTX on C2C12 and H9c2 was 0.8 mg/mL, and on A7r5 was at 0.2 mg/mL, respectively. Morphological changes as well as dead cells (arrows) were observed in A7r5 cell-treated with β-CTX. **(B)** Percentage of cell viability as function of toxin concentration (n = 3; three replicates for each n). Data are presented as mean ± S.E.M.

Prior to the cardiomyocyte functional studies, β-CTX was initially tested for its cytotoxicity assay on three different mammalian muscle cell lines, C2C12, A7r5 and H9c2. Microscopically images of β-CTX or PBS treated in different muscle cell lines were shown in Figure 2A. Morphological changes and cell death were observed (Figure 2A, arrows) in A7r5 myocytes (n = 3) after incubation with β-CTX at 0.2 mg/mL (28.57 µM). In contrast, there were neither morphological changes nor cell death observed in C2C12 cells and H9c2 at all tested concentration up to 0.8 mg/mL (114.09 µM) (Figure 2A). The cellular viability of β-CTX on A7r5 was assessed using the MTT assay and revealed 50% cytotoxic concentration (CC_50_) at approximately 0.07 ± 0.01 mg/mL (9.41 ± 1.14 µM) (Figure 2B). On the other hand, β-CTX had no effect on cellular viability in C2C12 and H9c2 up to 0.8 mg/mL.

### Effects of purified toxin on isolated cardiac myocyte function

**Figure 3.**
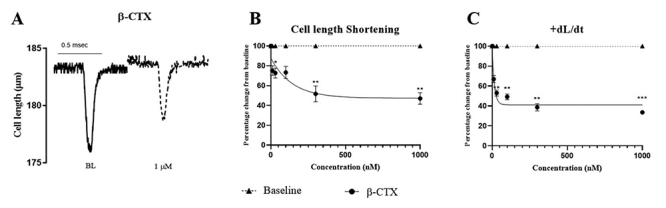
Effects of β-CTX on isolated cardiomyocyte inotropy. **(A)** Original recordings of cell length compared to baseline (BL) and following application of 1 µM β-CTX. Non-linear curve fits of **(B)** cell length shortening and **(C)** cell shortening velocity (+dL/dt) normalized to baseline data as function of toxin concentration. Data are represented as mean ± S.E.M. *p < 0.05; **p < 0.01; ***p < 0.001 vs baseline.

We sought to understand the effect of β-CTX on ventricular myocyte function by determining the dynamics of cardiomyocyte cell shortening and Ca^2+^ transient. The effect of β-CTX on cell length shortening is shown in Figure 3. Figure 3A shows a representative recording at baseline and in the presence of β-CTX, while Figure 3B shows normalized data as function of toxin concentrations recorded in all cells studied. Figure 3C summarizes normalized cell shortening velocity as function of toxin concentration (+dL/dt). Both the extent and rate of cell shortening were depressed by β-CTX, indicating a significant negative inotropic impact of the toxin. A non-linear curve-fit revealed that β-CTX inhibited the cell-length shortening and +dL/dt in a dose-dependent manner with the IC_50_ of 95.97 ± 50.10 nM and 10.23 ± 1.52 nM, respectively. 

**Figure 4.**
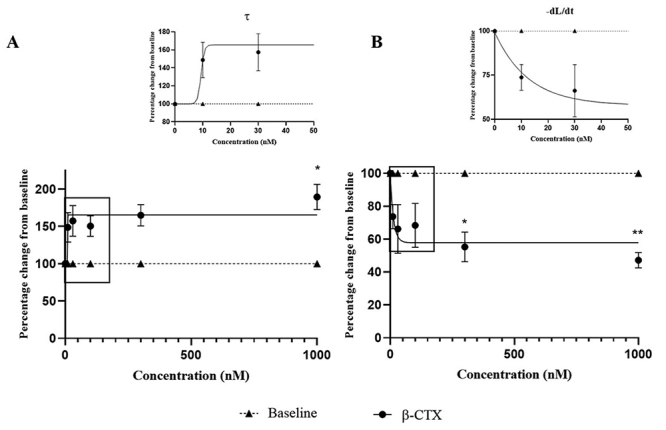
Effects of β-CTX on isolated cardiomyocyte lusitropy. Non-linear curve fits of **(A)** relaxation index (τ) and **(B)** re-lengthening velocity (-dL/dt) normalized to baseline as function of toxin concentration. Insets show data at an expanded scale from the boxed area to highlight data recorded at low concentrations of β-CTX. Data are represented in mean ± S.E.M. *p < 0.05; **p < 0.01 vs baseline.

**Figure 5.**
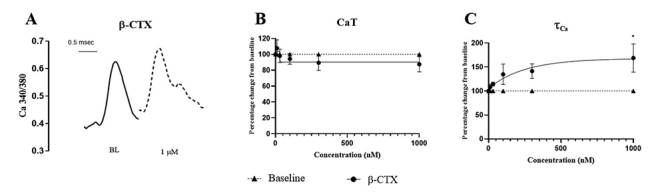
Impact of β-CTX on isolated cardiomyocyte calcium homeostasis. **(A)** Original recording of the intracellular calcium transient in an isolated cardiac myocyte before (BL) and after application of β-CTX (1 µM). **(B)** Non-linear curve fits of normalized peak calcium transients (CaT) and **(C)** calcium decay rate (τ_Ca_). Data are represented as mean ± S.E.M. *p < 0.05 vs baseline

The effects of β-CTX on cardiomyocytes diastolic function are presented in Figure 4A and 4B. β-CTX also significantly inhibited the lusitropic properties of the ventricular myocytes (*p* < 0.05) as indexed by the increase of the τ relaxation rate index (Figure 4A) and the reduction in re-lengthening velocity (-dL/dt) (Figure 4B) with an IC_50_ of 29.47 ± 12.74 nM and 22.91 ± 17.53 nM, respectively. The impact of β-CTX on the intracellular Ca^2+^ transient is summarized in Figure 5. Panel A shows a representative recording of intracellular Ca^2+^ as function of time during the twitch, while panels B and C show the normalized average data of the amplitude of the calcium transient (CaT; panel B) and rate of decay (τ_Ca_; panel C) as function of [β-CTX]. While β-CTX did not affect the amplitude of the calcium transient (up to 1 µM), it reduced the rate of decay (τ_Ca_) of the calcium transient in a dose dependent manner with an EC_50_ of 55.83 ± 13.67 nM. 

## Discussion

Initially, β-CTX was proposed as a novel β-blocker candidate based on isolated heart and *in vivo* studies. This is the first report to demonstrate the effect of β-CTX on cardiomyocyte function, Ca^2+^ homeostasis, and the cytotoxic effect of β-CTX on cultured skeletal, smooth and cardiac muscle cell lines. In addition, we present an alternative method to purify and isolate β-CTX from king cobra venom.

### Alternative isolation methods and characterization of β-CTX from Thai KCV

In the first report of β-CTX isolation, a two-step purification technique, gel filtration followed by reverse phase FPLC chromatography was used to obtain a purified compound [1]. In this study, we modified the purification protocol by using RP-HPLC as a first step. The procedure is similar to a previous proteomic study of the Indonesian’s king cobra venom [5]. Of note, our chromatographic profile is similar to this previous study. In addition, the elution time of the protein in our study (~60 minutes) is similar to that published method. However, mixtures of other 3FTX proteins, such as long-chain neurotoxin (LNTX; accession number P01387.1) and weak toxin (DE-1; accession number P01412.2) were also eluted from the first step column in this fraction. By comparing the theoretical isoelectric focusing point (pI) among these molecules, β-CTX displays the highest pI (8.85), followed by LNTX (8.05) and DE-1 (4.72). Hence, a cation exchange column separation using sodium phosphate as buffer at pH 7.4 was chosen for the second purification step. Indeed, pure β-CTX was isolated by the second chromatography as confirmed SDS-PAGE molecular weight estimation and the automated N-terminal amino acid sequencer. In term of cost-effectiveness, the method employed in the current study may provide benefits as compared to the previous FPLC method.

Although β-CTX was detected from proteomic profiles of the venom of Thai king cobra [3], this is the first study to isolate β-CTX from the Thai snake. The compound purified from the Thai KCV was similar to the Malaysian king cobra [4]. However, the amount of the β-CTX contained in the venom appears to vary between regions, such as Indonesian (0.7% w/w), Hainan (0.8% w/w) and Guangxi (2% w/w) king cobras [4, 6]. Notably, the protein composition variation in king cobra venoms influence the toxicity and immunological properties of the venom [6, 7]. Apart from the geographical distribution, variation of the components in snake venoms were have also been attributed to other factors, including age-related effects, nutrition and seasonal changes [9]. Even though the 3FTXs family of proteins accounts for the most abundant component found in the king cobra venom, β-CTX was reported to be only a minor ingredient [5]. According to the gene bank database, there are five different genes encoding for other cardiotoxins (CTX 9, 14, 15, 21, 23) in the venom gland of the king cobra [1]. These proteins contain several amino acid residues different from β-CTX, yet still may have similar functions. Although we did not characterize those proteins in the current study, we speculate that those other additional fractions identified in this study may refer to different CTX proteins and further functional studies of additional fractions are certainly warranted. 

### Cytotoxic effects of β-CTX on rat smooth muscle cells, but not striated muscle lines

The *in vivo* toxicity in mice of β-CTX was previously reported the lethal dose at 100 mg/kg [1]. In the current study, we present the *in vitro* cell viability assay of β-CTX on all types of muscle cell lines. Interestingly, β-CTX showed cytotoxicity in rat smooth muscle, while no lethal effect on skeletal and cardiac myoblast cells at the tested concentrations was observed. It appears, therefore, that β-CTX selectively induces cytotoxicity in smooth muscle. Since both skeletal and cardiac muscle cells share similar structural components, we speculated that β-CTX may affect the protein components that are present exclusively in smooth muscle cells, resulted in cell toxicity. The full elucidation of the cellular mechanisms underlying this differential impact between smooth and striated muscle, however, requires further study. To date, there have been no reports into the cellular mechanisms underlying the cytotoxicity of this novel protein. However, other related cobra cardiotoxins display cytotoxicity in several types of cells including neuronal, endothelial, cardiac muscle, skeletal muscle and cancerous cells [10-13]. Among these cells, the major pathway causing cellular death is perforation at cellular and sarcolemma membranes, thereby interfering with Ca^2+^ homeostasis in the cytosol [14, 15]. However, a previous study suggests that β-CTX does not exert an ionophore activity as the compound contained fewer charges as compared to the other CTXs [2]. The absence of myoblast cytotoxicity of β-CTX may be explained by the lack of the appropriate receptor for β-CTX or that the effective concentration of β-CTX in the myoblast cells was not reached in our study. Nevertheless, the mechanism of cytotoxicity by β-CTX needs further study. 

### Impact of β-CTX on cardiomyocyte contraction

In the current study, we found that β-CTX depressed the contraction and the rate of myocyte shortening during each contraction. However, the peak intracellular calcium concentration was not affected, indication no blunting of the amount of calcium released by the internal storage organelle, the sarcoplasmic reticulum. This result indicates a Ca^2+^-independent negative inotropic effect of β-CTX. It is well known that intracellular Ca^2+^ concentration plays an important role in modulating myofilament dynamics and ventricular contractility [16]. Generally, β-blockers reduce the activity of adenylyl cyclase activity, the cAMP-PKA pathways and, hence, Ca^2+^ homeostasis [17]. However, since peak intracellular Ca^2+^ was not affected by the toxin, our results indicate that β-CTX may act directly on myofilament calcium responsiveness or, indirectly, via other non-β-adrenergic pathways. This notion may explain a previous study by Rajagopalan et al. [1] who reported an unchanged contractility index upon β-CTX application in the *ex vivo* isolated heart model, even though it appeared that the toxin can bind to β-adrenergic receptors. We speculated that the effects of β-CTX may not be mediated through a classical β-adrenergic pathway, but rather, through other cellular mechanisms and/or its direct effect on myofilament proteins. Regardless, the cellular pathways by which β-CTX mediates a negative inotropic effect warrants further investigation. 

In preliminary studies, we found that β-CTX appears to be a more potent negative inotropic agent than propranolol as indicated by a much lower IC_50_ (~96 nM for β-CTX versus ~8 μM for propranolol; data not shown). Apart from β-blockers, other drugs that contribute to negative inotropic effects include Ca^2+^ channel blockers (e.g. verapamil or diltiazem), Na^+^ channel blockers (such as quinidine, flecainide, or mexiletine) and myosin II ATPase inhibitors (notably, blebbistatin, N-benzyl-*p* -toluene sulphonamide; BTS, or 2,3 butanedione monoxime; BDM) [18-20]. Of note, latter compounds are the only group that blunt ventricular myocyte contractility without affecting the peak intracellular calcium concentration reached in during the calcium transient. Interestingly, some snake venom toxins may act as negative inotropic agents via different cellular mechanisms. For example, calciceptine, a 3FTX protein isolated from the black mamba (*Dendroaspis polylepis polylepis*) venom, dose-dependently inhibits rat atrial myocyte contractility and causes smooth muscle relaxation through a specific block of L-type Ca^2+^ channels (LTCC) [21]. Dendroaspis natriuretic peptide (DNP) also reduces Ca^2+^ influx through LTCC, resulting in reduced ventricular contraction [22]. Angusticeps-type toxins, peptides isolated from *Dendroaspis angusticeps*, also show a negative inotropic and chronotropic impact via inhibition of cholinergic receptors [23]. Our findings of a blunted cardiac contractility are consistent with a report on Taiwan cobra (*Naja oxiana*) derived toxin [24]. In contrast, however, other studies report positive inotropic properties of Elapidae derived CTX proteins [14, 25]. Hence, we can exclude the possibility that specific cardiac toxins (proteins) derived from various species display opposing cardiac phenotypes and underlying cellular mechanisms. 

### β-CTX induced prolonged Ca^2+^-decay and negative lusitropy

Lusitropic parameters measured in our current study reveal that β-CTX suppresses the rate of cell relaxation (τ parameter) and reduces the rate of cell re-lengthening (-dL/dt parameter), indicative of reduced ventricular relaxation and impaired diastolic function. It is likely that this impact of cellular mechanics is mediated by a decreased rate of Ca^2+^ uptake by the sarcoplasmic reticulum, as indicated by a prolongation of the decay of the intracellular calcium transient (τ_Ca_ parameter). The rate of calcium removal from the cardiac cytosol is controlled by the sarcoplasmic reticulum Ca^2+^ pump (SERCa; indirectly controlled by phospholamban), as well as Ca^2+^ efflux rates via the sarcolemmal Ca^2+^ pump and Na^+^/Ca^2+^ exchanger [26]. We speculated that β-CTX may interfere with the rate of calcium reuptake/efflux via these proteins, possibly via post-translational modifications. Consistent with this notion, there are reports on snake toxins that induce reduced cardiac lusitropy. For example, SRTX-i3 [27] and SRTX-m [28] were shown to blunt ventricular relaxation parameters in *in vivo* hemodynamic and echocardiographic studies, indicating impaired diastolic dysfunction of the heart. It should be noted that the molecular mechanisms underlying the action of these toxins to date have not been investigated. Nevertheless, it has been reported that administration of the β-adrenergic receptor blocking agent does not affect ventricular relaxation indices as evaluated by echocardiography [29] or *ex vivo* pressure-volume measurement [30]. In addition, the rate of intracellular Ca^2+^ decay in isolated ventricular myocytes is not affected upon application of propranolol [31]. These data strengthen our hypothesis that β-CTX may not act via the classical β-adrenergic signaling pathway. Rather, other possible cellular mechanisms by which β-CTX directly affects diastolic function include inhibition of SERCa or Na^+^/K^+^ ATPase. Taken together, we hypothesize that β-CTX may interact with multiple targets as has also been reported for other three-finger toxins [32]. For example, ρ-Da1a, from green mamba (*Dendroaspis angusticeps*), shows a binding affinity to both α_1_ and α_2_ adrenergic receptors [33]. Muscarinic toxins from elapidae also were reported to impact cardiac function via activation of type 2 or 3 muscarinic receptors [34]. Clearly, further studies possibly involving these aforementioned cellular mechanisms are needed to fully elucidate the molecular mechanisms of action underlying the cardiac impact of king cobra snake derived β-CTX. 

## Conclusion

We successfully purified β-CTX from the Thai KCV using sequential reverse-phase and cation exchange chromatography. We revealed a negative inotropic and negative lusitropic cellular impact of β-CTX in isolated cardiac myocytes. The blunted contractility in the absence of altered peak intracellular calcium concentration implies reduced myofilament calcium sensitivity, while reduced relaxation is likely caused by a prolongation of the calcium transient decayed. Further studies are required to elucidate the effects of β-CTX on non β-adrenergic signaling pathways as well as pathways mediated by other receptors are required to fully elucidate the mechanism of action of the snake venom derived toxin.

### Abbreviations

+dL/dt: shortening velocity; 3FTX: three-finger toxin; Ca^2+^: calcium; CaT: calcium transients; cIEx: cation exchange; CTX: cardiotoxin; -dL/dt: re-lengthening velocity; FPLC; fast protein liquid chromatography; HPLC: high performance liquid chromatography; KCV: king cobra venom; MTT: 3-(4,5-Dimethylthiazol-2-yl)-2,5-diphenyltetrazolium bromide; β-CTX: beta-cardiotoxin; τ: relaxing index; τ_Ca_: calcium-decaying time.
